# Exosomes in organ transplantation: roles in immunomodulation, ferroptosis and mitophagy

**DOI:** 10.3389/fimmu.2026.1725547

**Published:** 2026-06-02

**Authors:** Yunuo Jiang, Peiran Xu, Tianyun Gao, Chong Wang, Meng Fan, Zhantong Tang, Koulong Zheng, Pengyu Liu

**Affiliations:** 1 Department of Cardiology, Nantong First People’s Hospital, Southeast University, Nantong, China; 2 Department of Medical Genetics and Developmental Biology, Jiangsu Provincial Key Laboratory of Critical Care Medicine, School of Medicine, Southeast University, Nanjing, China

**Keywords:** exosomes, ferroptosis, immunoinflammatory response, mitophagy, transplantation

## Abstract

Exosomes are extracellular vesicles (EVs) with a lipid bilayer structure, ranging from 30 to 150 nm in diameter, that are secreted by cells. As key carriers of biological information, they play pivotal roles in diverse physiological and pathological processes, such as immune response, apoptosis, angiogenesis, and inflammation. Advancing research has revealed that exosomes transport immunomodulatory cargo relevant to transplantation, demonstrating their capacity to directly modulate immune rejection and tolerance, beyond merely serving as biomarkers for assessing graft function and acceptance. Consequently, exosomes present significant therapeutic potential in transplant immunology. Furthermore, ferroptosis and mitophagy, two burgeoning fields of research, are increasingly recognized to interact closely with exosomes and participate in coordinated pathophysiological processes. This review aims to summarize the characteristics of exosomes and elaborate on their roles, alongside ferroptosis and mitophagy, in organ transplantation, with a focus on their collective therapeutic implications.

## 1 Introduction

**Figure 1:**
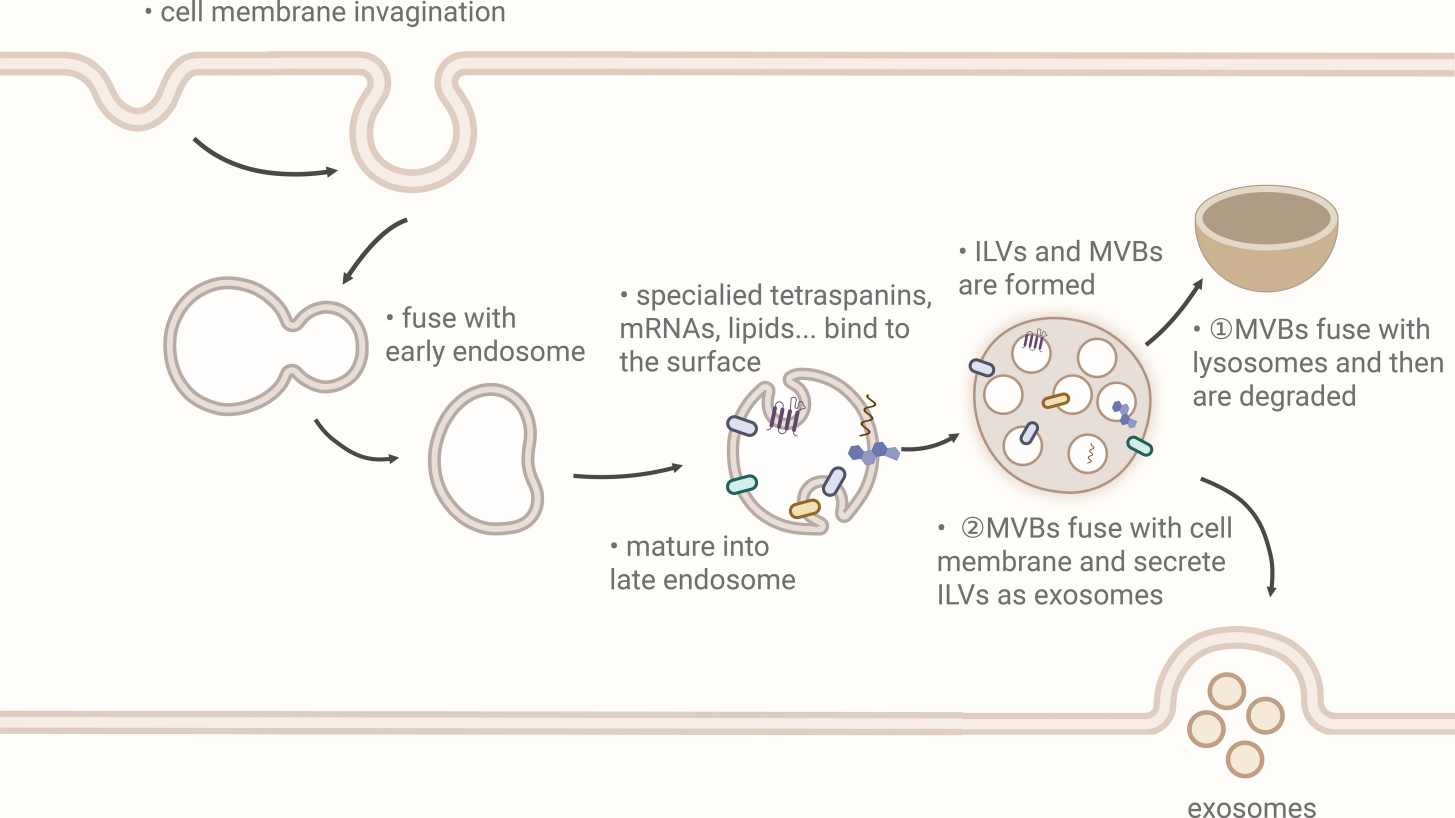
Schematic illustration of exosome biogenesis and release.

Exosomes, a distinct subclass of EVs secreted by nearly all cell types, are characterized by a phospholipid bilayer enclosure and a diameter typically ranging from 30 to 150 nanometers. Their biogenesis follows a unique endosomal pathway, which fundamentally distinguishes them from other vesicles such as apoptotic bodies and microvesicles that bud directly from the plasma membrane. The process initiates with the inward invagination of the endosomal membrane, leading to the formation of an early endosome. This compartment subsequently matures into a late endosome, which selectively incorporates diverse intracellular cargo — including proteins, lipids, and nucleic acids. Through further membrane invagination, the late endosome evolves into a multivesicular body (MVB), harboring numerous intraluminal vesicles (ILVs) within its lumen (1, 2). The subsequent trafficking of MVBs is intricately governed by the cytoskeleton. Following a critical fate-determination step, a subset of MVBs is destined for degradation via fusion with lysosomes, where the ILVs and their molecular contents are hydrolyzed. Alternatively, another subset is precisely transported to the plasma membrane. There, through specific interactions with SNARE family proteins, these MVBs fuse with the plasma membrane and release their ILV cargo into the extracellular space as exosomes through the process of exocytosis (2, 3). This endosomal pathway of exosome biogenesis is schematically illustrated in **Figure 1**.

**Figure 2:**
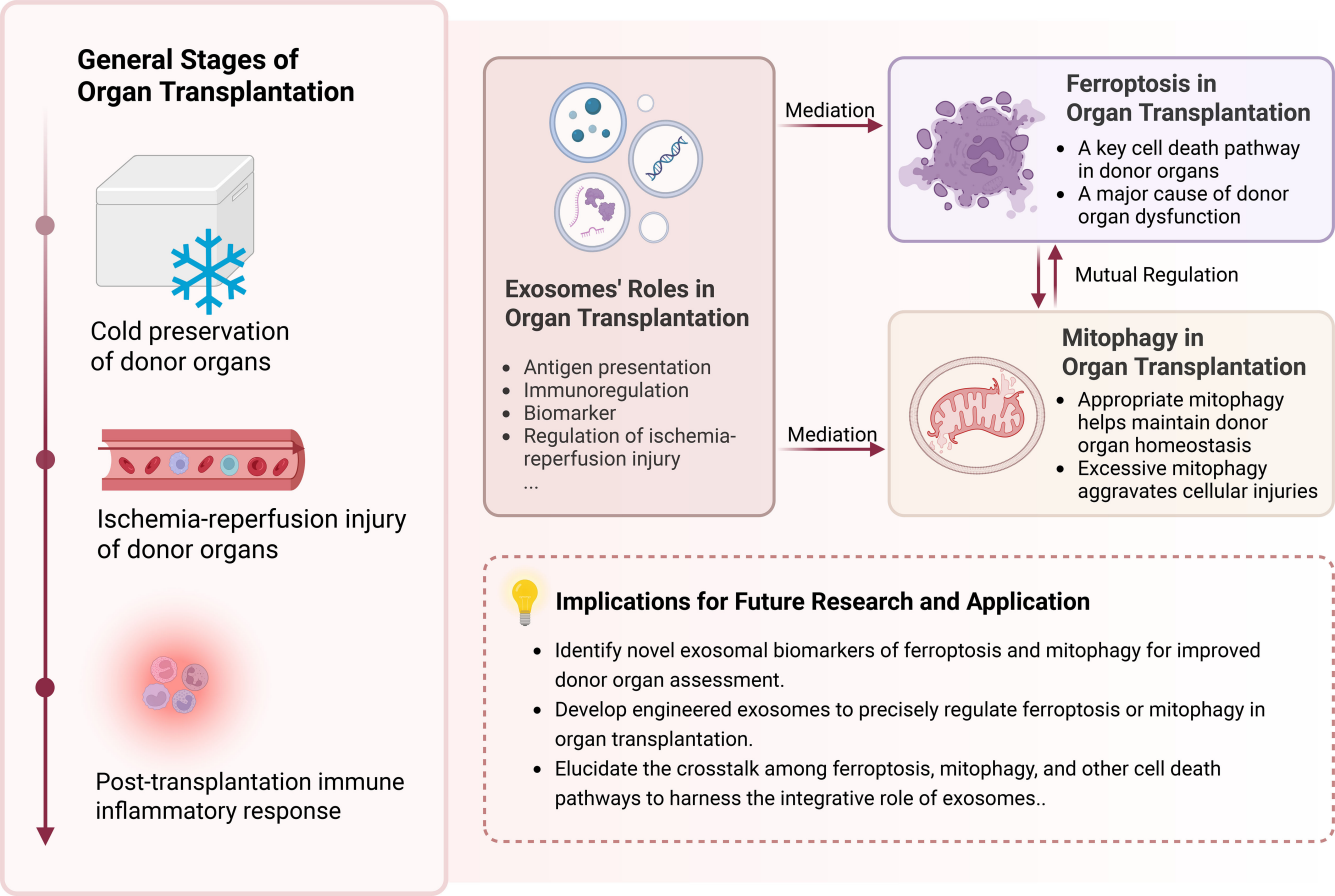
Diagram of general stages in organ transplantation: cold preservation, ischemia-reperfusion injury, and post-transplant immune inflammatory response.

The functional heterogeneity of exosomes is fundamentally determined by their diverse cellular origins, unique constituent profiles (including proteins, nucleic acids, and lipids), and consequent spectrum of biological activities (4). A key feature underpinning this heterogeneity is the exosomal membrane, which displays a repertoire of cell-specific antigens—including fusion proteins, adhesion molecules, and integrins—that dictate the specificity of recipient cell recognition and binding (5). These inherent properties, namely their biomarker potential and targeted delivery capability, form the foundation for their significant promise in clinical fields such as diagnostic, therapeutic, and drug delivery platforms (**Figure 2**). The process initiates with endocytosis, trafficking cargo to early endosomes, which mature into MVBs. MVBs subsequently fuse with the plasma membrane, releasing intraluminal vesicles as exosomes into the extracellular space.

## 2 Exosome-based therapeutics in organ transplantation

**Figure 3:**
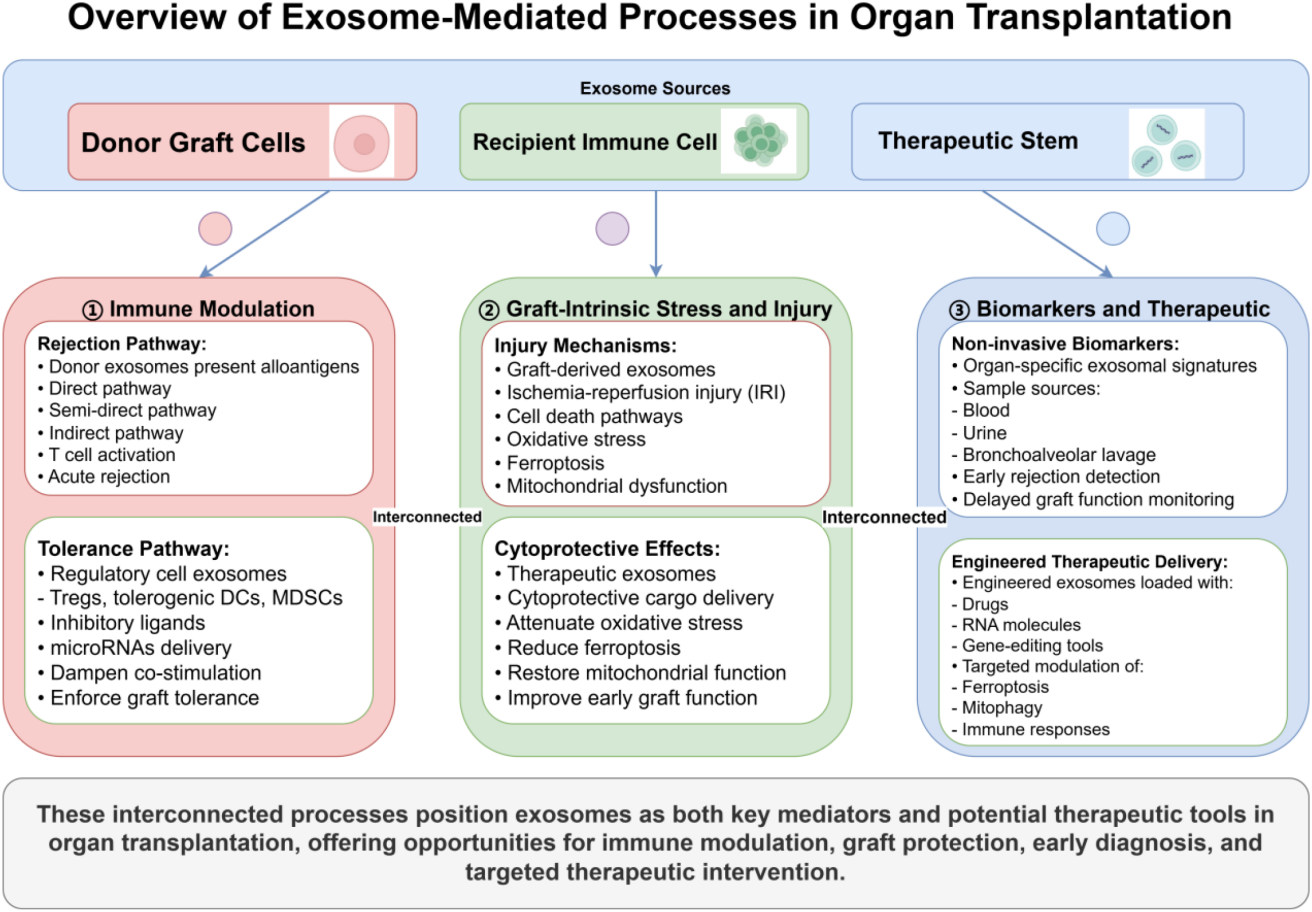
Overview of exosome-mediated processes in organ transplantation.

Exosomes regulate a myriad of biological processes—ranging from intercellular communication and cargo transport to tissue repair and cell death regulation—thereby influencing nearly all cellular states in both health and disease (6, 7). In the context of organ and tissue transplantation, exosomes are increasingly recognized as central mediators that link three key dimensions of transplant biology: (i) allogeneic immune recognition and the balance between rejection and tolerance, (ii) ischemia–reperfusion injury and graft-intrinsic stress responses, and (iii) non-invasive biomarker readouts and targeted delivery of therapeutic cargo. These multifaceted roles mean that exosomes can either exacerbate graft injury (e.g., by amplifying alloimmune activation) or support graft survival (e.g., by promoting immune regulation and tissue repair). In this section, we first summarize how exosome-mediated antigen presentation and immunomodulation shape transplant immunity, and then highlight how these mechanisms are being translated into exosome-based therapeutic and biomarker strategies. These multifaceted roles mean that exosomes can either exacerbate graft injury or support graft survival. An overview of these exosome-mediated processes in transplantation is illustrated in **Figure 3**.

### 2.1 Antigen-presenting properties of exosomes

**Figure 4:**
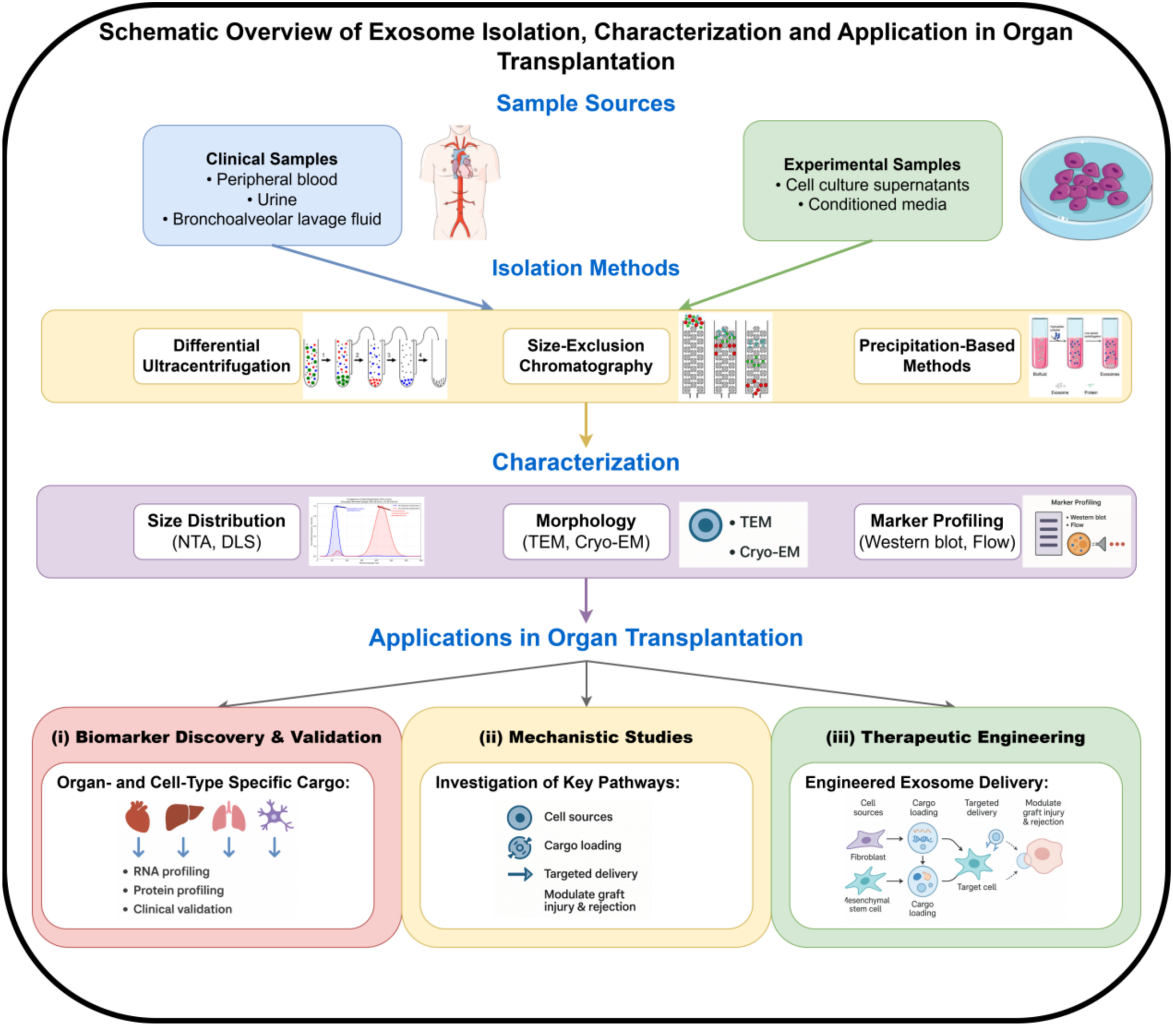
Workflow of exosome isolation, characterization and downstream applications.

Exosomes from different cell types have distinct immunomodulatory effects, which broadly fall into two categories—immunostimulatory and immunosuppressive—along a continuum that ultimately determines whether graft rejection or tolerance predominates. At the “pro-rejection” end of this spectrum, donor-derived exosomes participate in alloantigen presentation and T cell activation, whereas at the “pro-tolerance” end, exosomes from regulatory immune cell subsets enforce inhibitory signals and dampen effector responses, laying the mechanistic foundation for exosome-based interventions that aim to tilt the balance toward graft acceptance (**Table 1**). **Figure 4** provides an integrated workflow of exosome isolation, characterization and downstream applications, which underpins the therapeutic and mechanistic sections that follow.

**Table 1:** Summary of mechanisms and studies related to the antigen-presenting properties of exosomes.

Mechanism/study type	Exosome source	Core function	Key molecules/Cells	References
[Immunostimulatory]				
Direct Allorecognition	Donor APCs	Direct allorecognition via p-MHC-TCR binding	p-MHC, TCR, CD4+/CD8+ T cells, Donor APCs	(8–10)
Semi-Direct Allorecognition	Donor (exosomal MHC internalized by recipient APCs)	Recipient APCs present donor MHC to activate CD4+/CD8+ T cells	Donor MHC, Recipient APCs, CD4+/CD8+ T cells	(8, 9)
Indirect Allorecognition	Donor (exosomal MHC internalized by DCs/Macrophages)	DCs/macrophages present donor MHC via endogenous MHC pathway	Donor MHC, DCs, Macrophages, Endogenous MHC pathway	(10, 11)
Anti-Tumor Vaccine Model	Mature DCs (mDECs) with neoantigens	Promotes T cell differentiation and B cell activation	mDECs, Neoantigens, T cells, B cells	(12)
[Immunosuppressive]				
T-cell Apoptosis Induction	Immature DCs (imDECs) expressing FasL	Induces apoptosis of Fas-expressing T cells	imDECs, FasL, Fas, T cells	(19)
T-cell Inhibition	Tregs (Treg-exos)	Downregulates APC co-stimulatory molecules (CD80/CD86)	Treg-exos, CTLA-4, APCs, PD-L1, T cells	(20)
DC Function Impairment	Tregs (Treg-exos) with specific miRNAs	Inhibits DC antigen processing and presentation	Treg-exos, miR-150-5p, miR-142-3p, DCs	(21)

APCs, Antigen-Presenting Cells; CTLA-4, Cytotoxic T-Lymphocyte-Associated Protein 4; DCs, Dendritic Cells; imDECs, Immature Dendritic Cells; MHC, Major Histocompatibility Complex; miRNA, microRNA; m-DECs, Mature Dendritic Cells; PD-L1, Programmed Death-Ligand 1; TCR, T Cell Receptor; Treg-exos, Treg-Derived Exosomes; regs, Regulatory T Cells.

#### 2.1.1 Immunostimulatory effects

A key immunostimulatory function of exosomes lies in their antigen-presenting ability, primarily mediated through three allogeneic recognition pathways. First, exosomes derived from donor antigen-presenting cells (APCs) display MHC-antigen peptide complexes (p-MHC) on their surface; these complexes enable direct allogeneic recognition by binding to T cell receptors (TCRs) on CD4+ or CD8+ T cells (8–10). Second, in the semi-direct pathway, intact donor exosomal MHC complexes are internalized by recipient APCs, which then present these complexes via their own antigen presentation machinery, thereby activating both CD4+ and CD8+ T cells (8, 9). Third, indirect allogeneic recognition is initiated when donor exosomal MHC molecules are taken up by dendritic cells (DCs) or macrophages; these molecules subsequently undergo proteolytic processing and are presented via endogenous MHC pathways to boost immune activation (10, 11).

From the perspective of transplantation biology, these exosome-dependent antigen-presentation routes provide a mechanistic explanation for how donor-derived vesicles can prime and propagate anti-graft responses, thereby contributing to acute rejection. At the same time, they also illustrate why exosomes are attractive therapeutic targets: selectively blocking pathogenic exosomal antigen presentation or redirecting exosomal cargo toward tolerogenic pathways may mitigate alloimmune injury without globally suppressing host immunity.

Notably, exosomes exhibit potential in antitumor immunotherapy due to their immunostimulatory properties. It has been demonstrated that personalized antitumor vaccines—formulated with mature DC-derived exosomes (m-DECs) loaded with patient-specific neoantigens—effectively induce the differentiation of naïve T cells into cytokine-secreting CD4+ T cells and cytotoxic CD8+ T cells. Additionally, these vaccines activate B cell-mediated broad-spectrum immune responses, which significantly enhance cellular immunity in murine models (12). Although these studies were conducted in oncology, they conceptually highlight how exosome-based antigen delivery systems could also be engineered in transplantation to either enhance protective immunity (e.g., anti-infective responses in immunosuppressed recipients) or, conversely, be dampened to avoid anti-graft reactivity.

This dual potential is underscored in transplantation, where exosomes can mediate both allograft rejection and the induction of immune tolerance, depending on their cellular origin and cargo (13, 14). For instance, while donor-derived exosomes can prime anti-graft responses, exosomes from regulatory immune cells have been shown to promote graft acceptance (15, 16).

#### 2.1.2 Immunosuppressive effects

In contrast to mature DC-derived exosomes (which exert immunostimulatory effects), certain exosome subsets mediate immunosuppression to maintain immune homeostasis or prevent excessive immune responses. Specifically, it has been reported that immature DC-derived exosomes (im-DECs) expressing Fas ligand (FasL) selectively induce apoptosis in Fas-expressing T cells, thereby suppressing T cell-mediated immunity (17).

Furthermore, exosomes derived from regulatory T cells (Treg-exos) play a critical role in immunosuppression. It has been found that regulatory T cells (Tregs) and their secreted Treg-exos downregulate the expression of CD80/CD86 (co-stimulatory molecules on APCs that are essential for T cell activation) on APCs via cytotoxic T-lymphocyte-associated antigen-4 (CTLA-4)-dependent trogocytosis. This suppression of co-stimulatory signals, together with increased surface expression of PD-L1 (a molecule that inhibits T cell activation) on target cells, collectively inhibits T cell activation (18). Similarly, it has been identified that Treg-exos transfected with miR-150-5p and miR-142-3p (microRNAs (miRNAs) that regulate gene expression at the post-transcriptional level) impair DC functions—specifically by suppressing DC-mediated antigen uptake, proteolytic processing, and MHC-dependent antigen presentation—thus indirectly inhibiting downstream immune responses (19).

Taken together, these immunosuppressive exosome populations exemplify how vesicle-mediated mechanisms can actively enforce peripheral tolerance. For transplant immunology, they provide a biological blueprint for constructing tolerogenic exosome-based therapeutics—either by expanding endogenous Treg-exos or by engineering exosomes to deliver inhibitory ligands and miRNAs—to shift the immune balance from graft rejection toward long-term acceptance.

### 2.2 Immunomodulatory properties of exosomes

Exosomes exhibit dual immunomodulatory functions, and their inhibitory properties lay the foundation for the development of exosome-based therapies targeting transplantation-associated immune complications. Importantly, both their immunostimulatory and immunosuppressive activities can be viewed not only as pathogenic drivers of graft injury but also as therapeutic opportunities once the direction, magnitude, and cellular source of exosomal signals are understood and controlled.

Graft-versus-host disease (GvHD)—the primary complication following stem cell transplantation—arises from concurrent pathological processes: donor T cells recognizing recipient alloantigens and excessive activation of recipient-derived immune cells. For instance, it has been demonstrated that mesenchymal stem cell-derived exosomes (MSC-exos) attenuate natural killer (NK) cell-mediated immune responses in GvHD patients by suppressing the production of tumor necrosis factor-α (TNF-α) and interferon-γ (IFN-γ) (20). Building on this finding, it has been further revealed that MSC-exos carrying immunosuppressive factors (e.g., PD-L1 (21), transforming growth factor-β (TGF-β), and Galectin-3 (22)) inhibit the expression of co-stimulatory molecules on DCs, reduce DC cytokine secretion, and impair T cell proliferation and activation—ultimately alleviating the progression of GvHD (23).

Beyond MSC-derived exosomes, it has been identified that exosomes from rapamycin-treated myeloid-derived suppressor cells (MDSCs) deliver miR-181d-5p. This microRNA suppresses the nuclear translocation and expression of KLF6 (Krüppel-like factor 6), a downstream target, which in turn reduces inflammatory responses and alleviates transplant rejection (24). Such findings highlight a second layer of therapeutic potential, whereby exosomes are not only natural by-products of immunoregulatory cells but can also be “educated” by pharmacologic conditioning to become more potent tolerogenic vectors.

Collectively, these findings highlight exosomes as precision-engineered biological carriers capable of improving transplant outcomes through immunologically targeted interventions. Conceptually, the same vesicle-based mechanisms that underlie alloantigen presentation and immune activation (Section 2.1) can be redirected toward immune regulation, thereby transforming exosomes from passive markers of graft injury into active tools for inducing and maintaining transplant tolerance.

### 2.3 Mitigation of ischemia-reperfusion injury by exosomes

Ischemia-reperfusion injury (IRI) represents an unavoidable pathological process in clinical organ transplantation, in which initial hypoxic stress and microvascular dysfunction occurring during ischemia are exacerbated by subsequent reperfusion. This dual-phase injury not only enhances immunogenicity by upregulating the expression of MHC-II-like molecules and adhesion molecules on epithelial cells and DCs, but also promotes DC maturation via damage-associated molecular patterns (DAMPs) released from stressed graft cells—thereby potentiating adaptive immune responses and increasing the risk of rejection (33). Thus, strategies that attenuate IRI have direct implications for both early graft function and subsequent alloimmune responses.

Emerging evidence identifies exosomes as potent modulators of IRI across multiple organ systems. For neuronal protection, astrocyte-derived exosomes have been shown to deliver miR-361, which suppresses the AMPK/mTOR signaling pathway and inhibits apoptosis in cerebral IRI models (34). For renal preservation, exosomes derived from human amniotic epithelial cells (hAEC-exos) attenuate acute kidney injury by dual blockade of the TNF-α/MAPK and caspase cascades (35). For cardiac repair, bone marrow mesenchymal stem cell (BMSC)-derived exosomes (BMSC-exos) deliver lncRNA HCP5 to activate the IGF1/PI3K/AKT pro-survival pathway in myocardial IRI (36). For hepatic protection, BMSC-exos also deliver miR-25b-3p to mitigate hepatocyte necrosis in IRI by inhibiting the PTEN signaling pathway (37).

Although many of these studies have been conducted in preclinical models outside of the strict transplant setting, they converge on a common theme: exosomes can be used as vehicles to deliver nucleic acids and proteins that dampen cell death pathways, maintain microvascular integrity, and reduce inflammatory activation. Positioned alongside their immunomodulatory and biomarker functions, these cytoprotective properties reinforce the notion that exosomes are not merely passive bystanders in transplantation but versatile therapeutic agents with the capacity to modulate both graft-intrinsic injury and host immune responses in an integrated manner.

Furthermore, addressing the technical and practical challenges is paramount for the successful clinical translation of exosome-based therapies. Key hurdles include the development of scalable and standardized methods for exosome isolation and purification, as well as improving the reproducibility of exosome production and characterization for therapeutic applications (1, 6). The delivery of exosomes to target organs remains another significant challenge, requiring strategies to enhance their stability in circulation, improve targeting specificity, and reduce off-target effects (1, 5). From a clinical translation perspective, issues such as large-scale manufacturing, batch consistency, and the definition of appropriate dosing and administration routes still need to be resolved before exosome-based therapies can be broadly applied in transplantation (5, 6). Overcoming these challenges will be critical to harnessing the full therapeutic potential of exosomes in transplantation.

### 2.4 Biomarker characterization of exosomes

The tissue specificity of exosomes allows their molecular cargo in bronchoalveolar lavage fluid (BALF), urine, or peripheral blood to be utilized as diagnostic biomarkers for the early detection and therapeutic monitoring of transplant rejection. From a translational standpoint, this biomarker dimension is tightly coupled to the immunobiology described above: shifts in exosomal composition reflect dynamic changes in donor organ stress and the host immune response, and thus provide a non-invasive window into ongoing graft pathology or tolerance.

Circulating exosomes in peripheral blood have been shown to exhibit organ-specific diagnostic potential across various transplantation modalities. In cardiac transplantation, donor-derived exosomes have been demonstrated to display 91.4% sensitivity and 95.8% specificity for the detection of acute rejection (AR) (25). Beyond cardiac applications, significant upregulation of circulating miR-33a-5p, miR-98-5p, and miR-151a-5p has been observed in renal transplant recipients with delayed graft function (26). Similarly, islet-derived exosomal miR-29b-3p, miR-216a-5p, miR-375, and miR-148a-3p in peripheral blood have been found to correlate with the severity of hypoxic injury and inflammatory responses following islet transplantation (5).

Exosomes in BALF have been shown to possess diagnostic potential for complications of lung transplantation, even prior to the emergence of histologic evidence of rejection (38). Notably, genes including CXCL16, IL-33, and EEA-1—whose expression is elevated in stable BALF exosomal small RNA (esRNA) profiles—have been found to exhibit reduced expression during AR, thereby serving as potential biomarkers for the early detection of lung transplant rejection (27). Additionally, elevated levels of exosomal Kα1T and Col-V in BALF have been correlated with the risk of bronchiolitis obliterans syndrome (28, 29).

Urinary exosomes offer noninvasive monitoring for renal transplantation. The stability of their mRNA and quantifiable microRNA signatures (e.g., has-miR-21-5p, has-miR-31-5p, has-miR-4532) have been shown to enable accurate AR diagnosis (AUC = 0.93, compared with 0.57 for eGFR) and differentiation of rejection types (30–32). Together, these studies indicate that exosomal cargo profiles are not only mechanistically linked to graft injury and immune status but can also be translated into clinically actionable tools for risk stratification and therapy guidance. Representative exosomal biomarkers for organ transplantation are summarized in **Table 2**.

**Table 2:** Summary of exosomal biomarkers for organ transplantation.

Transplanted organ	Exosome source	Biomarker	Core function	References
Heart	Peripheral blood	Donor-derived exosomes	Detect AR (sensitivity 91.4%, specificity 95.8%)	(25)
Kidney (DGF)	Peripheral blood	Circulating miRNAs (miR-33a-5p, etc.)	Upregulated in recipients with DGF	(26)
Pancreatic islet	Peripheral blood	Islet-derived exosomal miRNAs (miR-29b-3p, etc.)	Correlate with hypoxic injury/inflammation post-transplant	(5)
Lung (early rejection)	BALF	esRNA-related genes (CXCL16, etc.)	High in stable state, low in AR (early rejection marker)	(27)
Lung (BOS risk)	BALF	Exosomal proteins (Kα1T, Col-V)	Elevated levels link to BOS risk	(28, 29)
Kidney (AR diagnosis)	Urine	Exosomal mRNA, miRNAs (has-miR-21-5p, etc.)	Diagnose AR (AUC = 0.93 > eGFR 0.57); differentiate rejection types	(30–32)

AR, Acute Rejection; AUC, Area Under the ROC Curve; BALF, Bronchoalveolar Lavage Fluid; BOS, Bronchiolitis Obliterans Syndrome; DGF, Delayed Graft Function; eGFR, Estimated Glomerular Filtration Rate.

Despite these promising findings, several limitations and challenges currently constrain the clinical translation of exosome-based biomarkers in transplantation. First, exosome isolation and characterization methods remain heterogeneous across studies (e.g., ultracentrifugation, size-exclusion chromatography, polymer-based precipitation), leading to variability in yield, purity and downstream measurements. This lack of methodological standardization hinders cross-study comparability and the establishment of robust diagnostic thresholds. Second, different biological fluids—peripheral blood, urine, bronchoalveolar lavage fluid and others—possess distinct background noise, co-isolated vesicle populations and soluble contaminants, all of which can dilute or obscure graft-specific exosomal signals.

A third challenge lies in distinguishing donor-derived from recipient-derived exosomes in clinical samples. In many settings, both sources coexist and dynamically change over time, making it difficult to ascribe specific biomarker signals to graft-intrinsic damage versus host immune activation. In addition, exosomal cargo composition is highly context-dependent and influenced by cell type, activation state, ischemia–reperfusion injury, infection and immunosuppressive regimens, resulting in marked biological heterogeneity. From a clinical laboratory perspective, there is still a lack of validated reference ranges, standardized reporting units and universally accepted cut-off values for exosomal mRNA, miRNA or protein biomarkers across different transplant populations.

Finally, implementation of exosome-based assays raises practical considerations, including the cost and technical complexity of isolation and sequencing platforms, requirements for rapid sample processing, and regulatory and ethical issues related to repeated sampling and long-term biobanking of vesicle-rich biofluids. Addressing these methodological, biological and logistical challenges will be essential before exosomal biomarkers can be widely adopted as routine tools for monitoring graft function and rejection risk.

## 3 Exosome-mediated ferroptosis in transplantation

Beyond their immunomodulatory functions described in Section 2, exosomes also regulate metabolic forms of cell death that critically influence graft outcomes. Among these, ferroptosis has emerged as a central determinant of cold preservation injury, ischemia–reperfusion injury and alloimmune-mediated tissue damage.

**Figure 5:**
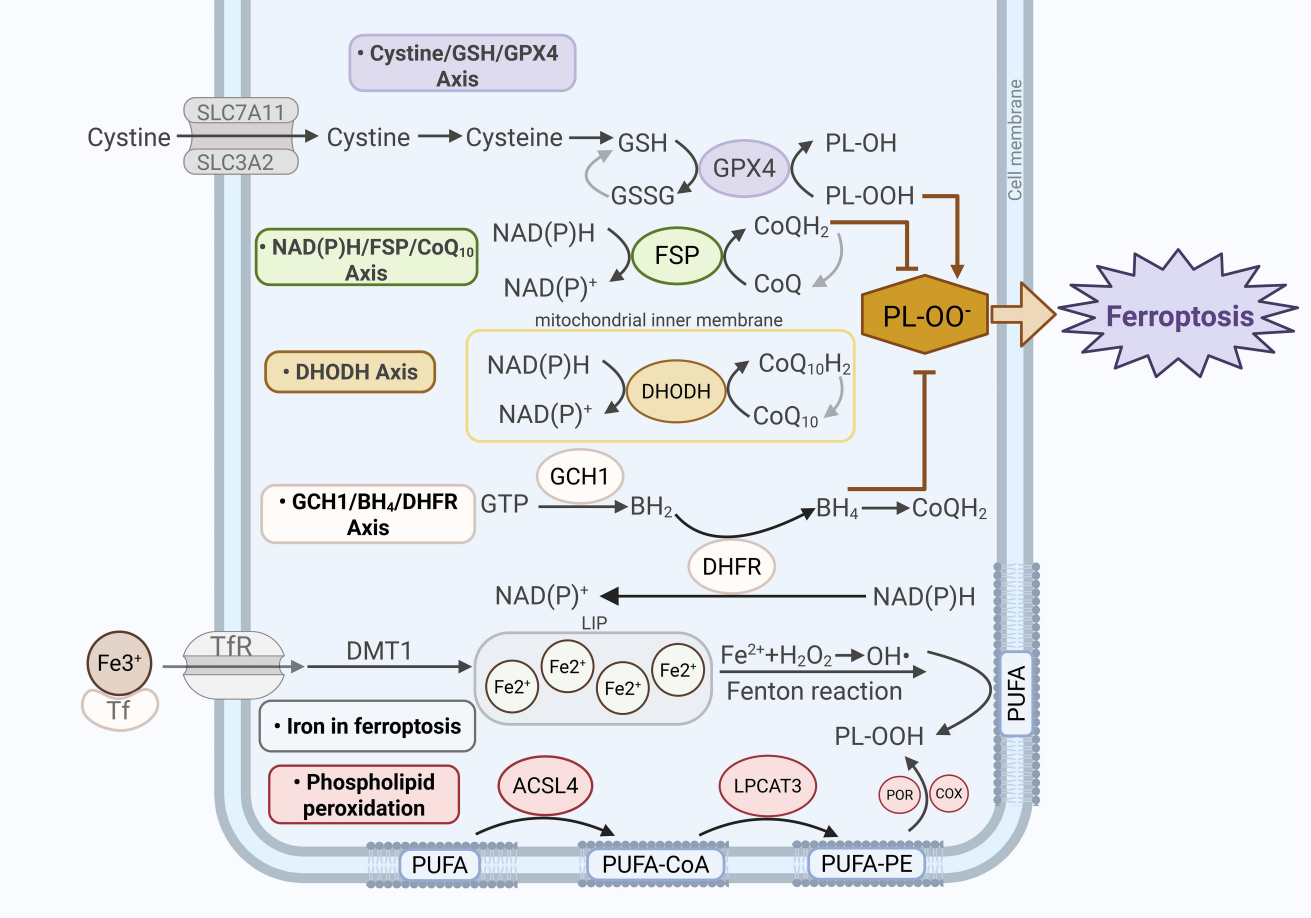
Molecular mechanisms of exosome-mediated regulation in ferroptosis.

Ferroptosis is an iron-dependent form of regulated cell death driven by excessive intracellular iron and unchecked lipid peroxidation (39). The key molecular mechanisms involved in ferroptosis are illustrated in **Figure 5**. Although multiple biochemical processes contribute to ferroptotic injury, two major pro-ferroptotic pathways are considered central: dysregulated iron metabolism, which amplifies ROS production and fuels lipid radical propagation (40, 41). and PUFA-phospholipid oxidation, in which oxidizable membrane lipids become substrates for lethal peroxidation (42).

Conversely, cells rely primarily on two anti-ferroptotic pathways to counteract lipid peroxidation: the glutathione–GPX4 axis, which detoxifies lipid hydroperoxides and preserves membrane integrity (43, 44), and several GSH-independent antioxidant systems, including the FSP1-CoQ10 and DHODH-coupled pathways, which suppress the accumulation of toxic lipid radicals (42, 45, 46).

From a transplantation perspective, the relevance of ferroptosis extends far beyond its biochemical definition. Cold ischemia and ischemia–reperfusion injury (IRI)—two unavoidable insults during organ procurement and implantation—are potent triggers of ferroptosis. Iron dysregulation, ROS burst, and membrane lipid oxidation occurring in these early phases not only exacerbate graft parenchymal and endothelial cell injury but also enhance graft immunogenicity, thereby predisposing organs to acute rejection. Consequently, ferroptosis has emerged as a mechanistic bridge linking metabolic stress, innate immune activation, and early graft dysfunction.

Exosomes add an additional layer of complexity and therapeutic opportunity to this process. With their ability to encapsulate and transfer regulatory RNAs, proteins, and lipids in a targeted and stable manner, exosomes can either reinforce ferroptotic signaling or blunt it, depending on their cellular origin and molecular cargo. These characteristics position exosomes as both active participants in ferroptosis-driven transplant injury and promising therapeutic vectors capable of delivering anti-ferroptotic cargo to vulnerable graft tissues. Representative mechanisms by which exosomes regulate ferroptosis in transplantation are summarized in **Table 3**. The following subsections summarize how exosome-mediated ferroptosis influences cold preservation, IRI, and immune-inflammatory responses in transplanted organs.

**Table 3:** Exosome-mediated ferroptosis regulation in transplantation.

Organ/context	Exosome source	Key cargo	Target pathway	Functional effect	Reference
[Liver]					
Steatotic Liver IRI	HO-1–modified BMSC	miR-29a-3p	IREB2	Inhibits ferroptosis, attenuates IRI	(61)
Steatotic Liver IRI	HO-1–modified BMSC	miR-124-3p	STEAP3	Suppresses ferroptosis, improves graft tolerance	(62)
Fatty Liver/Biliary IRI	BMSC	miR-204-5p	ACSL4	Reduces ferroptosis, alleviates biliary IRI	(63)
Cold/Septic Injury	ADSC	Nrf2/GPX4-activating cargo	Keap1–Nrf2–GPX4	Inhibits endothelial ferroptosis	(52)
Immune-Related Injury	MSC	SLC7A11-stabilizing cargo	System Xc–/GPX4	Reduces hepatocyte ferroptosis	(67)
[Kidney]					
Allograft IRI	IRI-derived sEVs	lncRNA WAC-AS1	GFPT1/HBP → BACH2	Propagates ferroptosis and graft injury	(56)
IRI/AKI	Urine Stem Cell (USC)	lncRNA TUG1	ACSL4	Inhibits ferroptosis, reduces AKI	(57)
Tubular IRI	Hypoxia-preconditioned Tubular Cells	miR-20a-5p	ACSL4	Suppresses ferroptosis, protects tubules	(58, 59)
Chronic Injury	Dental Pulp Stem Cell (DPSC)	Nrf2/GPX4 regulatory cargo	Nrf2–Keap1–GPX4	Inhibits ferroptosis and inflammation	(54)
[Lung]					
Acute Lung Injury	ADSC	miR-125b-5p	Keap1–Nrf2–GPX4	Inhibits endothelial ferroptosis	(69, 70)

### 3.1 Exosome-mediated ferroptosis in cold preservation

Cold ischemia exposes donor organs to hypoxia, metabolic suppression and oxidative stress, creating conditions that strongly predispose graft cells to ferroptosis (47, 48). During this phase, donor-organ–derived exosomes predominantly reflect injury-associated signaling rather than protective activity, whereas recipient- or therapy-derived exosomes introduced during storage or after reperfusion can exert active anti-ferroptotic effects. Evidence from liver and kidney transplantation indicates that endothelial and tubular epithelial cells undergo lipid peroxidation, glutathione depletion and GPX4 inhibition during preservation, collectively amplifying cold-storage injury (49–51).

Exosomes have emerged as key modulators in this setting. ADSC-derived exosomes activate the NRF2/GPX4 axis and attenuate ferroptosis in liver sinusoidal endothelial cells, while DPSC-exosomes exert similar protection in renal tubular cells by reinforcing the Keap1–Nrf2–GPX4 pathway (52). These findings highlight exosomes as promising anti-ferroptotic biologics with inherent targeting and low immunogenicity—advantages over broad ferroptosis inhibitors, whose clinical utility is constrained by poor specificity and off-target effects (53).

Thus, during cold preservation, exosomes function as cytoprotective vectors capable of mitigating ferroptosis-driven graft injury and improving organ quality prior to transplantation.

### 3.2 Exosome-mediated ferroptosis in Ischemia-reperfusion injury

Ischemia–reperfusion injury (IRI) is a universal challenge in transplantation, and ferroptosis is now recognized as a major driver of IRI-induced graft dysfunction. Exosomes released under ischemic stress can either propagate or suppress ferroptotic damage depending on their cellular origin and molecular cargo.

In kidney IRI, injury-associated sEVs enriched in WAC-AS1 promote GFPT1-mediated metabolic reprogramming and enhance ferroptosis (54), whereas urine-derived stem cell exosomes (USC-exos) and hypoxia-preconditioned tubular exosomes suppress ferroptosis through TUG1- or miR-20a-5p–mediated downregulation of ACSL4, consequently reducing AKI severity (55–57).

In steatotic liver grafts—highly vulnerable to IRI—HO-1/BMSC-derived exosomes deliver ferroptosis-inhibitory miRNAs (e.g., miR-29a-3p, miR-124-3p, miR-204-5p), targeting IREB2, STEAP3 or ACSL4 to alleviate hepatocellular injury and enhance graft performance (58–61).

Mechanistic insights from myocardial infarction models further support the concept that exosome-regulated ferroptosis shapes cardiac IRI: ferroptotic cardiomyocyte-derived exosomes polarize macrophages toward a pro-inflammatory state, whereas macrophage-derived exosomes deliver miR-155 to impair cardiomyocyte repair (62, 63). These observations are highly relevant to heart transplantation, where primary graft dysfunction parallels MI-associated IRI (62).

Collectively, exosomes act as upstream regulators of ferroptosis during IRI, offering a targeted approach to restrain ferroptotic injury and preserve graft function.

### 3.3 Exosome-mediated ferroptosis in immune inflammatory response.

Beyond cold preservation and IRI, ferroptosis also amplifies immune-mediated inflammatory injury after transplantation. Alloimmune activation—particularly IFN-γ secretion by CD8^+^ T cells—suppresses anti-ferroptotic pathways such as SLC7A11, thereby sensitizing hepatocytes and other graft cells to ferroptosis during rejection (64).MSC-derived exosomes counterbalance this effect by stabilizing SLC7A11 and reducing ferroptosis-associated liver injury (65).

In heart transplantation, ferroptosis promotes neutrophil accumulation and vascular endothelial damage through TLR4/TRIF/type I IFN signaling, whereas MSC-exosomal miR-125a-3p attenuates neutrophil activation and ameliorates inflammatory injury (62, 66).

Similarly, in lung transplantation, ADSC-exosomal miR-125b-5p activates the Keap1/Nrf2/GPX4 axis and inhibits ferroptosis in pulmonary endothelial cells, providing protection against acute lung injury (67, 68). The central role of GPX4 in T-cell proliferation and function underscores the need for precision targeting—exosome-based ferroptosis modulation must protect graft parenchymal cells without impairing adaptive immunity or inadvertently promoting rejection.

Together, these findings position exosomes as context-specific regulators of immune-inflammatory ferroptosis, capable of either propagating or resolving graft injury depending on their cargo profile.

## 4 Exosome-mediated mitophagy in transplantation

**Figure 6:**
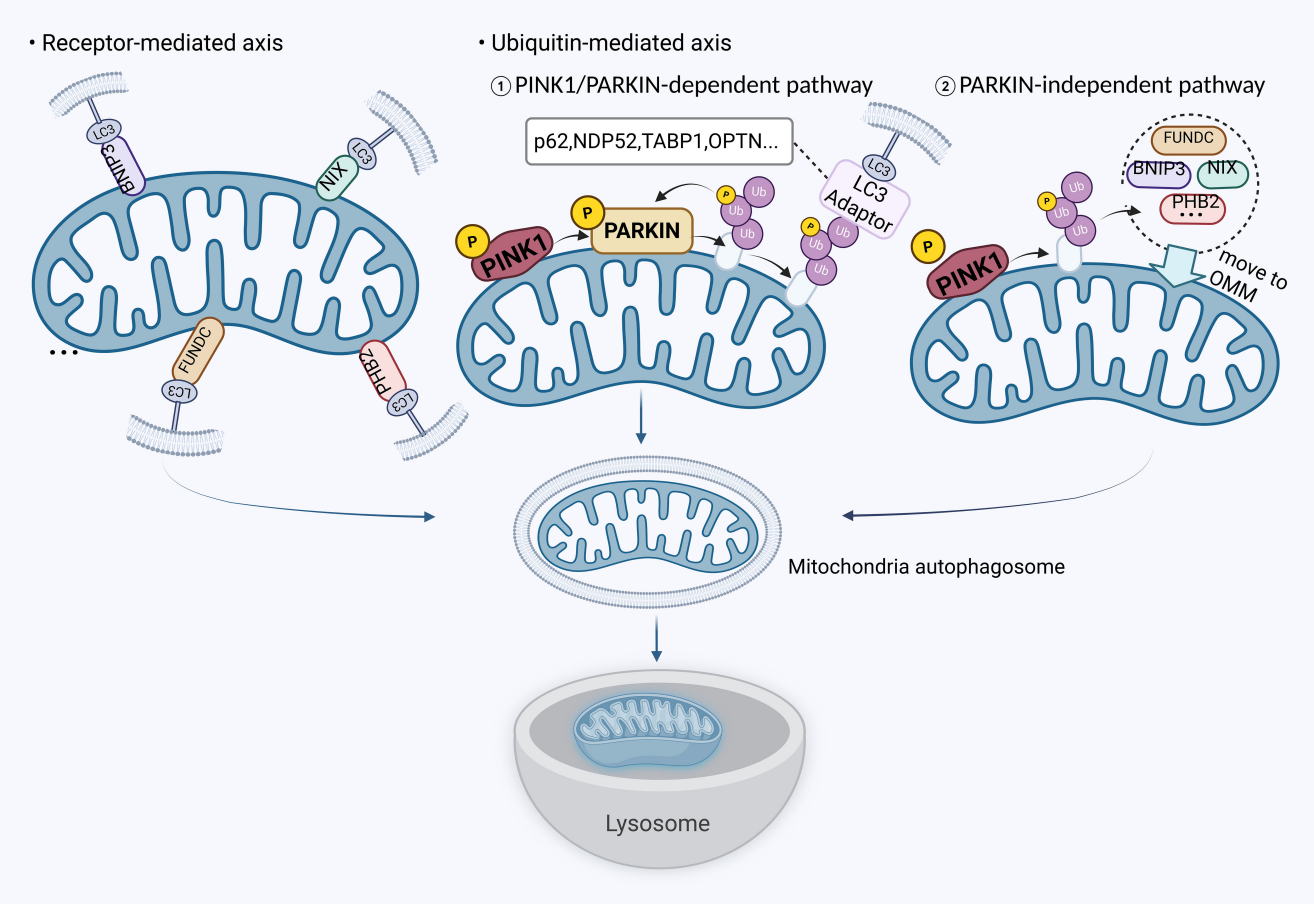
Receptor-dependent and ubiquitin-dependent mitophagy pathways.

Representative studies on exosome-mediated mitophagy regulation across transplantation contexts and tissue repair are summarized in **Table 4**. Mitophagy is a selective form of autophagy that removes damaged or dysfunctional mitochondria, thereby preserving mitochondrial integrity and maintaining metabolic homeostasis during stress conditions highly relevant to transplantation. Among multiple mitophagy mechanisms, three core pathways are most pertinent to graft injury and protection. The PINK1/Parkin pathway is activated by mitochondrial depolarization and oxidative stress, initiating ubiquitin-dependent clearance of dysfunctional mitochondria and limiting ROS accumulation during ischemia–reperfusion injury (69–72). The BNIP3/NIX axis, strongly induced by hypoxia, mediates receptor-dependent mitophagy and promotes elimination of damaged mitochondria in metabolically stressed graft tissues (73, 74). The outer mitochondrial membrane receptor FUNDC1 represents another major hypoxia-responsive mediator, linking environmental oxygen tension to adaptive mitochondrial turnover and cytoprotection (75–77). Together, these three pathways—PINK1/Parkin, BNIP3/NIX and FUNDC1—form the central mitophagy network through which transplanted organs respond to ischemia, inflammation and immunologic injury. The major receptor-dependent and ubiquitin-dependent mitophagy pathways are summarized in **Figure 6**.

**Table 4:** Exosome-mediated mitophagy regulation in transplantation.

Organ/context	Exosome source	Cargo	Mitophagy pathway	Functional effect	Reference
[Kidney]					
IRI/AKI	IL-10 EVs	IL-10	PINK1/Parkin via mTOR suppression	Enhances mitophagy, protects tubules	(85)
IRI	MSC	miR-223-3p	BNIP3-related, NLRP3 blockade	Reduces renal IRI	(87)
Septic AKI	Fibroblastic Reticular Cell	CD5L	PINK1/Parkin	Promotes mitophagy, reduces inflammation	(95)
[Heart]					
IRI/TX	VEC	SPC	NR4A2–OPTN/Parkin	Increases cardiomyocyte mitophagy	(88)
Post-TX Protection	Skeletal Muscle	Irisin	PTEN/PINK1/Parkin & FUNDC1	Reduces oxidative stress & inflammation	(96–98)
[Liver]					
Fatty Liver TX	Lipotoxic Hepatocyte	miR-27a	PINK1 suppression	Activates HSCs, increases inflammation	(93)
IRI	Hepatocyte Signaling	—	PINK1/Parkin	Suppresses NLRP3, reduces inflammation	(94)
[Tissue Repair]					
General Fibrosis	ADSC	Multiple regulators	p62–LC3–linked	Anti-fibrotic, anti-inflammatory	(99)
Bone/Cartilage	BMSC	Drp1 regulators, circHIPK3	Drp1 & PINK1/Parkin	Protects chondrocytes, enhances osteogenesis	(100, 101)
Diabetic Bone	Engineered MSC	SHP2	ER–mitophagy coupling	Maintains homeostasis, improves regeneration	(102)

Recent findings further demonstrate that exosomes intersect with these mitophagy pathways and can either enhance or inhibit mitochondrial turnover depending on their cellular origin and molecular cargo. The following subsections summarize how exosome-mediated mitophagy contributes to graft preservation, ischemia–reperfusion injury, immune inflammation and post-transplant tissue repair.

### 4.1 Exosome-mediated mitophagy in cold preservation

In organ transplantation, the role of mitophagy during cold preservation has attracted increasing attention. Immediate cooling of the donor heart—a critical initial step—exerts a significant impact on graft function. Recent evidence indicates that prompt cooling, compared with the inevitable 10–15 minutes of warm ischemia in standard procedures, enhances targeted cardiac protection by activating mitophagy (78). This process eliminates damaged mitochondria and preserves cellular homeostasis, thus highlighting mitophagy as a potential therapeutic target in heart transplantation.

The function of mitophagy during cold preservation may also interact with other stress-response pathways. Under cold ischemia-hypoxia, key regulators such as NRF2 become dysregulated (49). Notably, NRF2—a critical modulator of ferroptosis—is also closely associated with autophagy. Dysfunction of NRF2 enhances mitochondrial calcium uptake, which is correlated with the activation of mitophagy. Although direct evidence in the context of transplant cold preservation remains limited, insights from related hypoxia-ischemia models have revealed a dual role of mitophagy: moderate mitophagy mediated by the PINK1/Parkin pathway eliminates damaged mitochondria and exerts a protective effect (79), whereas its excessive activation may lead to energy depletion and cell death (80).

Exosomes represent a potential strategy for modulating mitophagy by targeting pathways such as the PINK1/Parkin signaling pathway. Specifically, human neural stem cell (hNSC)-derived exosomes and umbilical cord mesenchymal stem cell (UC-MSC)-derived exosomes have been shown to alleviate tissue injury through this pathway (81, 82). However, the safe and effective translation of such targeted approaches to organ cold preservation remains a key challenge.

### 4.2 Exosome-mediated mitophagy in Ischemia-reperfusion injury

In IRI, excessive accumulation of ROS and mitochondrial homeostasis imbalance are key factors that induce apoptosis and necrosis. Activation of mitophagy effectively maintains mitochondrial functional homeostasis, alleviates oxidative stress responses, and thereby mitigates IRI.

Exosomes were utilized by Tang et al. to deliver interleukin-10 (IL-10), which specifically targets tubulointerstitial macrophages and tubular epithelial cells. By inhibiting the mammalian target of rapamycin (mTOR) signaling pathway, these exosomes promote mitophagy, maintain mitochondrial health, and significantly improve tubular injury and inflammatory responses induced by renal IRI (83). This finding provides a potential therapeutic strategy for acute kidney injury following kidney transplantation.

Additionally, the NLRP3 inflammasome can inhibit BCL2/adenovirus E1B 19 kDa protein-interacting protein 3 (BNIP3)-mediated mitophagy (84). MSC-exos, which are rich in miR-223-3p, were further demonstrated by Sun et al. to directly target and inhibit NLRP3, thereby blocking inflammasome activation, enhancing mitophagy, and ultimately mitigating ischemia-reperfusion-induced renal injury (85). These studies provide novel therapeutic insights into mitigating IRI via targeted regulation of mitophagy.

During heart transplantation, IRI of donor cardiomyocytes significantly accelerates cardiomyocyte apoptosis. Vascular endothelial cell (VEC)-derived exosomes (VEC-Exos) have been revealed by research to activate the Parkin and nuclear receptor subfamily A group 2/optineurin (NR4A2/OPTN) pathway through the delivery of sphingosine-1,2,3,5-tetraphosphorylcholine (SPC). This mechanism enhances mitophagy levels in cardiomyocytes within an I/R mouse model, thereby reducing I/R-induced cardiomyocyte apoptosis (86).

In hepatic ischemia-reperfusion injury research, pterostilbene (PT) has been demonstrated by studies to inhibit mitophagy via inducing increased expression of mitophagy markers (LC3II/I, PINK1, and Parkin), thereby alleviating hepatic ischemia-reperfusion injury (87). It has also been found that PT reduces mitochondrial reactive oxygen species (mtROS) levels and attenuates NLRP3 inflammasome activation (88). Given PT’s potential role in regulating mitophagy, how to effectively deliver it to target organs warrants further consideration. Regarding PT delivery methods, combining incubation (IN) with microwave-assisted methods (MB) has been suggested by research to enhance the loading efficiency of PT-carrying exosomes (89). This novel delivery system offers a potential intervention strategy for tissue protection following organ transplantation.

### 4.3 Exosome-mediated mitophagy in immune inflammatory responses

Fatty livers used as donor organs are prone to immune rejection, a phenomenon associated with abnormal activation of hepatic stellate cells (HSCs). Activated HSCs secrete proinflammatory factors (e.g., MIP-2, MCP-1, TGF-β1, IL-6), exacerbating post-transplant immune-inflammatory responses (90). Notably, miR-27a-enriched exosomes from lipotoxic hepatocytes (lipotoxic HC-exos) are specifically taken up by HSCs. By downregulating PINK1 and its mediated mitophagy pathway, miR-27a promotes HSC activation (91)—providing new insights into donor immune inflammation in fatty liver transplantation.

Additionally, early inflammatory responses during hepatic IRI are driven by proinflammatory cytokines (e.g., IL-1β) from Kupffer cells. Reduced PINK1-mediated mitophagy enhances hepatocellular NLRP3 inflammasome activation, promoting IL-1β release and exacerbating post-I/R inflammation (92). Thus, preventing PINK1 downregulation in hepatocytes is a key target to ameliorate post-transplant inflammation.

Beyond the liver, exosomes regulate immune inflammation by modulating mitophagy in other organs. Exosomes from fibroblastic reticular cells (FRC-Exos) rich in CD5L act on primary kidney tubular cells (PKTCs), promoting mitophagy via PARKIN phosphorylation and PINK1/PARKIN activation—thus suppressing PKTC inflammatory responses and informing post-kidney transplant immune regulation (93). Skeletal muscle cell-derived exosomes containing irisin modulate mitophagy via multiple mechanisms: irisin clears damaged mitochondria in cardiomyocytes via the PTEN/PINK1/Parkin pathway (94), activates the AMPK/mTOR pathway to enhance mitophagy (95), and triggers FUNDC-mediated mitophagy (96)—collectively reducing cardiomyocyte oxidative stress and inflammation. Notably, autologous irisin-containing exosomes, with their high biocompatibility and low immunogenicity, show unique potential for myocardial protection after cardiac transplantation.

### 4.4 Exosome-mediated mitophagy in post-transplant organ and tissue repair

Following organ transplantation, inflammatory responses and tissue ischemia can trigger immune-mediated repair mechanisms, leading to scar tissue formation. Overactivation of immune cells and excessive scar proliferation may impair transplanted organ function. Adipose-derived stem cell-derived exosomes (ADSC-exos) have been shown to activate mitophagy by inhibiting the PI3K/AKT/mTOR signaling pathway and enhancing p62-LC3 interaction, thereby reducing inflammation and fibrotic responses in keloid fibroblasts (97). This mechanism offers a novel approach for organ protection during post-transplant repair.

Promoting bone regeneration after bone grafting is critical for accelerating tissue healing, improving surgical success, and enhancing patient outcomes. BMSC-exos promote bone regeneration through multiple mechanisms: On one hand, BMSC-exos specifically upregulate mitophagy-related protein expression (LC3-II/LC3-I, Beclin-1) in damaged chondrocytes, thereby inhibiting chondrocyte apoptosis (98); On the other hand, circHIPK3 carried by BMSC-exo targets and binds miR-29a-5p to upregulate PINK1 expression, activating PINK1/PARKIN pathway-mediated mitophagy and promoting osteoblastic differentiation of MC3T3-E1 cells (99). Notably, in diabetes-related bone regeneration, MSC-exos deliver SHP2 to promote mitophagy, effectively reducing intracellular ROS levels and maintaining endoplasmic reticulum homeostasis, thus creating a favorable microenvironment for bone regeneration (100). These findings not only provide novel therapeutic insights for post-bone-transplant tissue repair but also reveal an exosome-mediated mitophagy mechanism that may inform tissue repair research in other organ transplants.

Collectively, these findings illustrate that exosome-mediated mitophagy is not limited to cytoprotection but also contributes to long-term graft remodeling and tissue repair, providing a mechanistic counterbalance to ferroptosis-driven injury described in Section 3.

## 5 The development prospects and potential applications of exosome-mediated ferroptosis or mitophagy

Building on the mechanistic insights from Sections 2–4, the following subsections discuss how these pathways can be exploited for biomarker development and therapeutic engineering.

### 5.1 Application prospects of ferroptosis- or mitophagy-related exosomal biomarkers in organ transplantation

Although EVs contents in body fluids have been widely investigated as biomarkers for the early prediction and diagnosis of post-transplant rejection, research on exosomal ferroptosis- or mitophagy-related molecules as biomarkers remains relatively scarce—representing a promising yet underdeveloped field.

Preliminary evidence supporting this potential has emerged from recent studies. For instance, Akirin1 has been identified as a ferroptosis-promoting protein that enhances TP53-mediated downregulation of SLC7A11. Elevated Akirin1 expression in urinary exosomes from kidney transplant recipients at 48 hours post-transplantation has been shown to hold significant value for the early diagnosis of delayed graft function (DGF) (101), which supports its potential as a clinical diagnostic biomarker. Additionally, elevated levels of urinary mitochondrial DNA (UmtDNA) have been demonstrated to play a critical role in the early diagnosis of acute kidney injury (AKI) (102). Unlike free UmtDNA in urine (which is prone to degradation), exosome-encapsulated UmtDNA is protected from degradation and may serve as a reliable biomarker for predicting mitochondrial dysfunction, renal injury, and the progression/prognosis of kidney disease in the future.

Given the tight link between post-transplant pathological damage and ferroptosis/mitochondrial dysfunction, the exploration of exosomal ferroptosis- or mitophagy-related molecules as biomarkers carries substantial scientific value and broad clinical application prospects—laying the foundation for non-invasive, early diagnosis of transplant-related complications.

### 5.2 Prospects of ferroptosis- or mitophagy-targeted engineered exosomes in organ transplantation

Endogenous exosomes have been shown to alleviate post-transplant tissue damage by inhibiting ferroptosis or activating mitophagy, but their clinical application is limited by insufficient targeting ability and poor dose controllability. Engineered exosomes, by contrast, offer advantages such as tunable drug loading and traceable distribution—making them a more promising therapeutic tool for transplant medicine.

Currently, exosome-mediated regulation of ferroptosis/mitophagy (a frontier in genetic and cellular engineering) remains in the early stages for modulating donor organ cell death and promoting damage repair. Key advances in this field include the following:

First, viral-derived EMVs engineering: Non-enveloped viruses (e.g., Coxsackievirus B, CVB) have been shown to induce host cells to secrete EMVs containing viral components. These EMVs evade adaptive immunity (due to the absence of typical viral surface antigens), and their biological properties are linked to dynamin-related protein 1 (DRP1)-dependent mitophagy activation (103). Engineered complexes are formed by fusing *in vitro*-inactivated, modified EMVs with highly biocompatible exosomes; these complexes can precisely target mitochondrial quality control in transplanted organ cells, offering a novel strategy to improve graft survival and long-term patient outcomes.

Second, gene editing-exosome hybrid systems: A liver-targeted gene therapy approach using hepatocyte-derived exosomes for CRISPR-Cas9 RNP delivery has been proposed, which enables precise knockout of ferroptosis-promoting genes (e.g., ACSL4, LPCAT3) in donor hepatocytes (104). This technology enhances gene editing efficiency via the inherent targeting ability of exosomes while avoiding the immunogenicity associated with viral vectors. SIn a non-transplant osteoarthritis model, nano-engineered carriers have been constructed by fusing HEK293-derived exosomes with liposomes; when combined with a CRISPR activation system, this platform targets the Foxo3 gene, activates the PINK1/Parkin mitophagy pathway, and stabilizes mitochondrial function in chondrocytes (105). Although this study does not directly provide a transplantation therapy, it demonstrates an exosome-based CRISPR delivery platform capable of modulating mitophagy-related genes and offers a conceptual framework for future strategies targeting donor organs and graft tissues (105). These findings validate the dual advantages of gene editing-exosome combinations in regulating ferroptosis and mitochondrial homeostasis.

Third, exosomal modification techniques: Advances in techniques such as electroporation, transfection, co-incubation, and ultrasonic encapsulation (105)—which have already been applied in stroke and cancer research (106, 107)—have enabled precise engineering of exosomal membrane ligands. These modified exosomes can specifically recognize transplanted organ cells and target key nodes in ferroptosis (e.g., GPX4, ACSL4) or mitophagy (e.g., PINK1/Parkin pathway), thereby maintaining cellular homeostasis.

Collectively, these engineered exosome strategies provide practical tools for the targeted regulation of ferroptosis/mitophagy in transplanted organs, bridging the gap between basic research and clinical translation.

### 5.3 Prospects of exosome-mediated integrated regulation of ferroptosis and mitophagy in organ transplantation

Ferroptosis and mitophagy are both tightly intertwined with intracellular iron metabolism and oxidative stress, and their interactive regulation—rather than independent action—is pivotal for sustaining cellular homeostasis. This interactive regulatory network represents a more impactful yet understudied frontier in transplant medicine.

#### 5.3.1 Interactive mechanisms between ferroptosis and mitophagy

Emerging evidence has delineated that ferroptosis and mitophagy are closely related to ROS, share common regulator molecules and engage in crosstalk via intricate molecular networks, with their effects dictated by biological context (e.g., specific signaling pathway, disease phenotype, injury phase). Two principal interaction modalities have been characterized:

Figuratively speaking, the intense process of intracellular ferroptosis can be likened to “a room catching fire”. Here, ROS act as an accelerant; the higher their concentration, the fiercer the blaze, ultimately leading to cellular demise. Mitophagy, in this analogy, serves as a diligent “firefighter”. Appropriate levels of mitophagy promptly clear damaged mitochondria and abnormal proteins, preventing the accumulation of harmful substances like ROS, thereby “quenching the flames” to a certain extent and protecting the cellular “room” from complete collapse. Consequently, the general idea is that mitophagy exerts an inhibitory role in ferroptosis in most cases. Take BNIP3 for instance. BNIP3-mediated mitophagy has been shown to diminish mtROS levels, thereby conferring protection against ferroptosis (108). Furthermore, BNIP3 can also activate the p62-KEAP1-NRF2 signaling axis, which modulates ferroptosis-relevant pathways (e.g., DHODH/FSP1-CoQ10-NADH) to preserve intracellular iron homeostasis and redox equilibrium (109).

**Figure 7:**
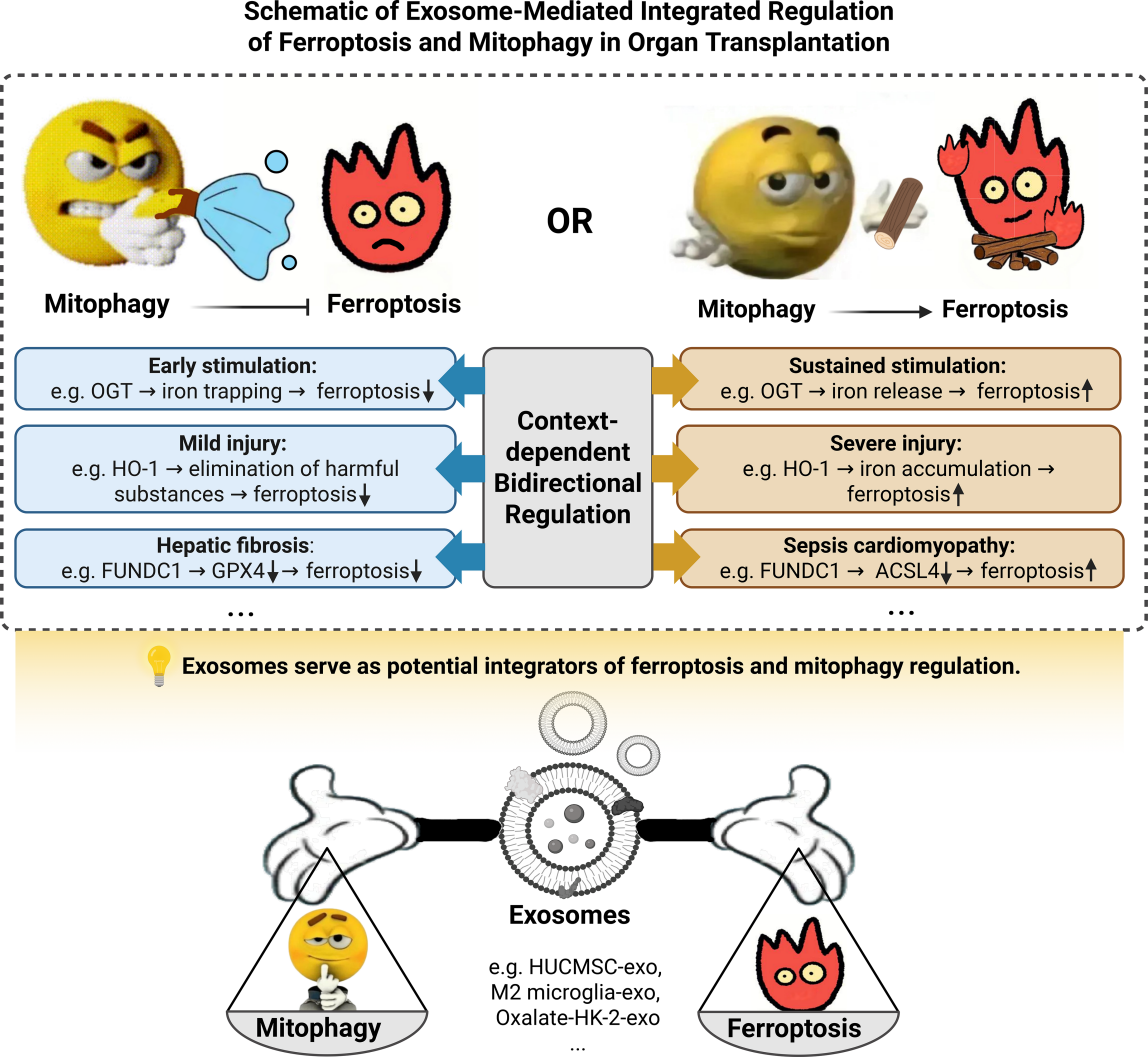
Schematic of exosome-mediated integrated regulation of ferroptosis and mitophagy.

However, mitophagy does not always play a firefighting role. Under certain conditions, the process of mitophagy may recruit factors that promote ferroptosis, effectively “adding fuel to the fire” and becoming an accomplice that exacerbates ferroptosis. Therefore, notably, context-dependent bidirectional regulation constitutes another key interaction pattern. First, the role of mitophagy may vary depending on the disease context. For example, in hepatic fibrosis, the mitophagy receptor FUNDC1 has been found to recruit cytoplasmic GPx4 to mitochondria via the TOM/TIM complex—a process that accelerates GPx4 degradation and subsequent ferroptosis (110). By contrast, in sepsis-induced cardiomyopathy, FUNDC1 directly abrogates ACSL4 activity, mitigating lipid peroxidation and thereby suppressing ferroptosis (111). On the other hand, the function of mitophagy may also differ according to the extent of tissue injury. For heme oxygenase-1 (HO-1), it promotes mitophagy to clear damaged mitochondria under conditions of mild injury; however, excessive HO-1 expression amid severe injury drives iron accumulation, ultimately triggering ferroptosis (112). Additionally, O-linked N-acetylglucosaminyltransferase (OGT) impedes the interaction between ferritin and NCOA4 during the early phase of ferroptosis stimulation, trapping iron within mitophagosomes to attenuate ferroptosis. Conversely, sustained ferroptosis stimulation precipitates mitophagosome-lysosome fusion, releasing sequestered iron to fuel ferroptosis-associated Fenton reactions (113). The context-dependent bidirectional relationship between mitophagy and ferroptosis, as well as the potential integrative role of exosomes, is illustrated in **Figure 7**.

#### 5.3.2 Exosomes as integrators of ferroptosis and mitophagy regulation

A central challenge in mitigating post-transplantation injury lies in the coordinated modulation of the interplay between ferroptosis and mitophagy. Harnessing their synergistic potential requires strategies that can simultaneously target both processes to restore cellular homeostasis. Herein, exosomes, with their low immunogenicity and inherent targeting specificity, emerge as a promising avenue for achieving this integrated regulation.

While the application of exosomes in co-regulating ferroptosis and mitophagy has not yet been explored in transplantation, a growing body of evidence from studies on other diseases supports this integrative role: For one thing, human umbilical cord mesenchymal stem cell (HUCMSC)-derived exosomes have been demonstrated to elicit activation of PINK1/Parkin-mediated mitophagy—a process that reduces programmed cell death (including ferroptosis) in the context of traumatic brain injury (114). For another, exosomes derived from oxalate-treated HK-2 cells have been shown to harbor Beclin-1. This molecule induces both mitophagy and ferroptosis in normal HK-2 cells, thereby contributing to the pathogenesis of kidney stone formation (115). Furthermore, M2 microglia-derived exosomes activate AMPK/ULK1 pathway-mediated FUNDC1-dependent mitophagy. This regulatory mechanism alleviates ferroptosis in OGD/R-induced HT-22 neurons, furnishing insights into the therapeutic management of neonatal hypoxic-ischemic brain damage (HIBD).

Therefore, translating these mechanistic insights into transplantation models represents a critical next step for developing novel therapeutic strategies.

#### 5.3.3 Research gaps and future directions

Current investigations predominantly center on exosomal regulation of either ferroptosis or mitophagy in the transplant setting, while systematic inquiries into exosome-mediated integration of their interactive networks remain scarce. Future research ought to focus on three pivotal directions:

1. Elucidate the mechanisms by which exosomes coordinate ferroptosis, mitophagy, and other cell death modalities (e.g., pyroptosis, cuproptosis, apoptosis) within transplanted organs;
2. Characterize context-specific regulatory paradigms (e.g., injury stage, organ type) governing exosomal integration of ferroptosis and mitophagy;
3. Develop multi-target intervention strategies grounded in these interactive mechanisms to more effectively mitigate graft injury.

## 6 Conclusion

Exosomes, as pivotal mediators of intercellular communication, do not regulate ferroptosis or mitophagy independently; rather, they play central roles in organ transplantation by integrating immune modulation, ferroptosis control, and mitophagy-mediated cytoprotection. By simultaneously attenuating oxidative stress, limiting lipid peroxidation, preserving mitochondrial homeostasis, and reshaping immune-inflammatory responses, exosomes emerge as multi-layered regulators of graft injury and repair. This coordinated regulation forms a more comprehensive protective network than single-pathway intervention and highlights exosomes as promising tools for precision diagnosis and targeted therapy in transplantation.

Importantly, compared with cell-based therapies such as mesenchymal stem cells (MSCs), exosome-based therapies may offer several translational advantages. As a cell-free therapeutic platform, exosomes are less likely to introduce risks associated with living-cell administration, such as uncontrolled proliferation, ectopic engraftment, vascular occlusion, or viability-dependent heterogeneity. In addition, exosomes may be easier to store, engineer, and standardize for targeted delivery. However, these advantages should be interpreted cautiously, because exosome-based therapies still face important safety concerns, including cargo heterogeneity, off-target biodistribution, contaminating co-isolates, and batch-to-batch variability. Therefore, the future clinical value of exosomes will depend not only on mechanistic efficacy, but also on rigorous safety evaluation and manufacturing standardization.

Another major challenge lies in defining and measuring the therapeutic dose of exosomes. In contrast to cell therapies, which are typically dosed by viable cell number, exosome dosing cannot yet rely on a single universally accepted metric. More practical approaches may require combined reporting of particle number, total protein content, source-cell equivalent, key cargo abundance, and functional potency assays. Establishing standardized dose metrics, potency criteria, and administration protocols will be essential for comparing studies and advancing exosome-based interventions toward clinical application.

Future research should therefore focus on three major directions: first, identifying exosomal cargo signatures linked to ferroptosis and mitophagy for early diagnosis and monitoring of graft injury; second, developing engineered exosomes with dual-target capacity to inhibit ferroptosis while fine-tuning mitophagy; and third, clarifying the context-dependent molecular networks through which exosomes coordinate these pathways across different transplanted organs. Overall, leveraging exosomes as core carriers and ferroptosis–mitophagy crosstalk as a regulatory framework may provide a new avenue for more precise and safer management of transplant injury and rejection.
